# Assessing the Feasibility of In Vitro Assays in Combination with Biological Matrices to Screen for Endogenous CYP450 Phenotype Biomarkers Using an Untargeted Metabolomics Approach—A Proof of Concept Study

**DOI:** 10.3390/metabo15120791

**Published:** 2025-12-12

**Authors:** Yannick Wartmann, Lana Brockbals, Thomas Kraemer, Andrea E. Steuer

**Affiliations:** Department of Forensic Pharmacology and Toxicology, Zurich Institute of Forensic Medicine, University of Zurich, Winterthurerstrasse 190/52, 8057 Zurich, Switzerland

**Keywords:** (un)targeted metabolomics, LC-(HR)MS, enzyme assay, in vitro, phenotyping, biomarker

## Abstract

Background/Objectives: Cytochrome P450 (CYP) enzymes are crucial for drug metabolism, yet inter-individual variability in their activity remains a significant clinical challenge. Current phenotyping methods are often impractical or even impossible, particularly in forensic toxicology and vulnerable populations. This proof-of-concept study investigated the feasibility of using in vitro assays with human liver microsomes (HLM) and recombinant CYP enzymes (isoenzymes), combined with untargeted metabolomics, to identify potential endogenous biomarker candidates indicative of CYP phenotype. Methods: This study uses in vitro incubations of HLM and isoenzymes in tandem with targeted and untargeted LC-(HR)MS and metabolomics techniques as well as statistical processing. Results: We demonstrate that HLM and isoenzymes maintain activity in the presence of complex biological matrices (blood/plasma), enabling metabolomic profiling. Untargeted analysis of assays in plasma revealed numerous potential biomarkers, with several showing significant correlations to enzyme activity. Conclusions: While identification remains the major challenge, this approach offers a promising avenue for developing accessible and efficient methods for indirect CYP phenotyping, potentially facilitating investigations in scenarios where traditional approaches are limited. This work provides a foundation for future studies focused on further developing in vitro assays and validating the proposed biomarkers, as well as establishing their utility in clinical and forensic settings.

## 1. Introduction

Cytochrome P450 (CYP) enzymes are vital in drug metabolism, where they account for about 75% of phase I-dependent drug metabolism. Of all clinically used drugs undergoing phase I metabolism, 70–80% are processed by CYP families 1–3. These enzymes mediate hydroxylation, oxidation, dealkylation, epoxidation, and dehalogenation [1,2,3]. Despite the well-established role, inter-individual variation in CYP activity remains a major challenge in pharmacology and particularly in forensic toxicology.

The actual function of the enzyme, known as the phenotype, is shaped by genetic polymorphisms, drug interactions, environmental factors, and physiological states. However, genotype-phenotype correlations are weak for many CYP families [4]. Current phenotyping relies on administering probe substrates and metabolite tracking, a method that demands extensive resources, time, and patient cooperation. Cocktail approaches improve efficiency but remain infeasible in forensic toxicology [5,6,7]. This is due to the fact that cases in forensic toxicology include subjects unwilling or unable to undergo extensive testing. Furthermore, forensic toxicology prioritizes rapid analysis and blood samples are typically collected as close to the time of the incident as possible. A phenotyping performed significantly after a forensic incident would be largely irrelevant to the investigation’s timeline and for identifying potential irregularities in drug metabolism at the time of the incident. Ultimately, the current gold standard approach is also not suitable in post-mortem toxicology [3].

Beyond forensic contexts, the current gold standard method is also limited in vulnerable populations such as children and pregnant women who may be unable to safely undergo testing with certain probe substrates. A reliable endogenous biomarker panel would enable non-invasive, timely CYP phenotyping.

Discovering reliable endogenous biomarkers indicative of a CYP phenotype would allow us to solve this persisting issue. Ideal markers should reflect enzyme activity without interference from external factors. A multi-marker or ratio-based approach is likely needed. Promising candidates include arachidonic acid and endogenous polyunsaturated fatty acid (EETs) for CYP2C19, 5-methoxytryptamine and serotonin as well as solanidine metabolites for CYP2D6, or cortisol and 6β-hydroxycortisol among other steroids for CYP3A4 [3]. Yet most face critical limitations. For example, some are urine-specific, often requiring long collection windows such as 24 h. Others are poorly studied in humans or lack isoform specificity or can be confounded by circadian rhythm or exogenous sources [3,8,9]. These challenges underscore the need for new biomarkers in forensic matrices like plasma or whole blood.

In recent years, metabolomics has become a widely applied tool to study endogenous compounds. While targeted metabolomics requires prior knowledge of pathways, untargeted approaches enable discovery of yet unknown metabolites linked to biological processes. Untargeted approaches, particularly in blood or plasma, have identified markers for drug use, sample tampering, and disease. Some are already validated and in clinical use [10,11]. These studies typically use in vivo samples, most often blood (plasma), collected after a stimulus, as multiple biological processes likely are affected, leading to widespread metabolite changes. Regarding CYP activity and function, for instance, Cheng et al. successfully evaluated the CYP2D6-related neurophysiological metabolism and function in an in vivo study in mice [12]. Magliocco et al. could identify solanidine metabolites to be a potential semi-quantitative CYP2D6 activity biomarker in humans [9]. Kim et al. proposed a model consisting of urinary ω- or (ω-1)-hydroxylated medium-chain acylcarnitines, 6β-hydroxycortisol and gender to predict midazolam clearance in male and female subjects and hence CYP3A phenotype [13]. However, ethical-, legal-, cost-, and complexity issues, such as the natural occurrence of, e.g., rare CYP enzyme phenotypes, limit the possibility of human in vivo studies. To overcome these issues, in vitro models offer a viable alternative.

One in vitro approach could be the use of human liver microsomes (HLM) and/or relevant isoenzymes that readily metabolize xenobiotics. These in vitro assays are frequently used in industry to test for drug–drug interactions (DDIs) [14]. They have also been used to screen for unknown metabolites of, e.g., new psychoactive substances (NPS) [15,16]. In all instances, a single or a few known substrates are added to the assay and their metabolites or effect on the enzymes are studied thereafter. Such assays have not yet been systematically applied to untargeted metabolomics experiments. To identify novel endogenous biomarkers, a broad range-or ideally the entire spectrum-of endogenous metabolites must be introduced. Since such a mixture is highly complex and commercially available standards are lacking, other options need to be explored. An accessible alternative is to use a comprehensive human matrix, such as blood or plasma, as a source of endogenous metabolites. A precedent for using recombinant enzymes with complex mixtures exists, as recombinant CYP7A1 has been applied to liver tissue extracts in combination with isotope-labeling strategies to identify novel endogenous substrates, such as 7α-hydroxycholesterol, by Dodda et al. [17] This confirms the feasibility of basic untargeted in vitro screening.

Therefore, our proof-of-concept study aimed to determine whether plasma can serve as a viable source of endogenous metabolites in HLM- and isoenzyme-based incubations. The primary aim was to demonstrate that HLMs and isoenzymes retain functional activity in complex biological matrices. We further tested whether a standard untargeted metabolomics workflow could be applied to such incubations, enabling the identification of potential novel endogenous CYP phenotype biomarkers for subsequent validation in smaller, more efficient in vivo studies.

## 2. Materials and Methods

### 2.1. Materials

Gentest^®^ HLM 150 Pools and isoenzymes (CYP2D6, CYP3A4, and CYP2C19) were purchased from Discovery Life Sciences (Huntsville, AL, USA). Glucose-6-phosphate (G6P, from baker’s yeast), glucose-6-phosphate dehydrogenase (G6PD, from baker’s yeast), super oxide dismutase (SOD, bovine recombinant from *E. coli*), and β-nicotinamide adenine dinucleotide phosphate (NADP+, hydrate) were purchased from Sigma Aldrich (St. Louis, MO, USA). Dextromethorphan, dextrorphan, midazolam, 1-hydroxymidazolam and cortisol were purchased from Cerilliant (Redrock, TX, USA). Omeprazole, serotonin, deoxycholic acid, arachidonic acid, and 5-methoxytryptamine were purchased from Sigma Aldrich (St. Louis, MO, USA). 11,12-Epoxyeicosatrienoic acid (11,12-EET) was obtained from Biogems (Westlake Village, CA, USA), 14,15-Epoxyeicosatrienoic acid (14,15-EET), 11,12-dihydroxy-eicosatrienoic acid (11,12-DiHET), 14,15-dihydroxy-eicosatrienoic acid (14,15-DiHET) were obtained from Cayman (Ann Arbor, MI, USA) and 5-hydroxyomeprazole from TRC (Toronto, Canada) and from Sigma Aldrich (St. Louis, MO, USA). Ammonium acetate (Chromasolve™) was purchased from Honeywell (Charlotte, NC, USA), ammonium formate (LiChropur™), methanol (OPTIMA^®^), and acetonitrile (ACN) from Sigma Aldrich (St. Louis, MO, USA). Acetic acid and formic acid were obtained from Biosolve Chimie (Dieuze, France). Water was either purchased from VWR Chemicals (Radnor, PA, USA, high resolution mass spectrometry) or taken from an in-house water purification system from Labtec (Villmergen, Switzerland). K2 ethylenediaminetetraacetic acid (EDTA, K2E) Vacutainer^®^ tubes were purchased from BD (Eysins, Switzerland). All other chemicals used were from Sigma Aldrich and of the highest grade available.

### 2.2. Blood and Plasma Samples

Whole blood was collected from one female volunteer into 6 mL K2E EDTA (10.8 mg) Vacutainer^®^ tubes (BD, Eysins, Switzerland). The volunteer was abstinent from substances that could influence assays or measurements, such as caffeine (more than 1 year) or any relevant drugs (at least more than 1 month). The volunteer was healthy and without chronic diseases or regular medication. All incubates originated from that same plasma batch. Plasma was prepared immediately after the blood was drawn by pipetting aliquots of blood into Falcon tubes and spinning them down at 2000 g for 10 min. The resulting plasma was immediately transferred and aliquoted into Eppendorf tubes before being stored at −20 °C. Whole blood was stored in aliquots in the same way.

### 2.3. In Vitro Assays

CYP in vitro incubation assays (CYP reaction) were performed according to standard practice [18]. Briefly, each assay (total volume of 50 µL) consisted of phosphate buffer (100 mM, pH 7.4), a co-substrate mix consisting of NADP+ (12 mM), G6P (50 mM), G6PD (5 U/mL), MgCl2 (50 mM) and buffer, SOD (200 U/mL), and HLM (1 mg/mL) or respective isoenzymes (CYP2D6, CYP2C19, CYP3A4, 10 pmol/mL, each). Depending on the different experiments, the buffer was (partly) replaced by 2 µL of an exogenous probe substrate mix (dextromethorphan, midazolam, and omeprazole, either 10 µM or 100 nM) and/or varying amounts (3.3–33 µL) of plasma or whole blood, representing an endogenous substrate mix. In addition, two negative controls were performed: negative control 1 (no enzyme, Neg1) and negative control 2 (no co-substrate, Neg2).

Samples were then shaken and incubated for 60 min at 37 °C. Afterwards, the reaction was stopped and protein precipitated by the addition of 50 µL of ice-cold ACN. Finally, 50 µL of the supernatant was transferred into an LC-MS vial and analyzed by targeted (see Section 2.4) and/or untargeted (see Section 2.5) analysis.

### 2.4. Targeted LC-MS/MS Data Acquisition

Semi-quantitative measurements were carried out on a Thermo Scientific Dionex UltiMate 3000 UHPLC system (Thermo Fischer Scientific, Waltham, MA, USA) coupled to a Sciex TripleQuad 5500 (Sciex, Framingham, MA, USA) using a scheduled MRM method with two transitions per analyte. Samples were measured in polarity-switching (electrospray ionization (ESI) positive and negative) mode with a flow rate of 0.5 mL/min on a XSelect^®^ HSS T3 2.5 µm, 2.1 × 150 mm XP column (Waters, Wexford, Ireland). The column oven was set to 40 °C, and the injection volume to 2 µL. Eluent A was a 10 mM ammonium formate buffer with 0.1% (*v*/*v*) formic acid in water, and eluent B was methanol with 0.1% (*v*/*v*) formic acid. The gradient (total run time 21 min) was 0–1 min 25% B, 1–2.6 min to 28% B, 2.6–15 min to 100% B, 15–18 min hold, 18.1 min return to start, 21 min end. The following MRM transitions (precursor > product, *m*/*z*) were monitored: dextromethorphan, 272 > 213 and 272 > 171; dextrorphan, 258 > 199 and 258 > 157; midazolam, 326 > 291 and 326 > 249; 1-OH-midazolam, 342 > 324 and 342 > 203; omeprazole, 346 > 149 and 346 > 168; 5-OH-omeprazole, 362 > 214 and 362 > 196; serotonin, 177 > 160 and 177 > 115; 5-methoxytryptamine, 191 > 174 and 191 > 130; cortisol, 363 > 121 and 363 > 91; 6-hydroxycortisol, 423 > 347 and 423 > 205; arachidonic acid, 303 > 259 and 303 > 205; 11,12- EET, 365 > 167 and 365 > 179; 11,12-dihydroxy-eicosatrienoic acid (DiHET), 337 > 167 and 337 > 169; 14,15-EET, 365 > 219 and 365 > 257; and 14,15-DiHET, 337 > 207 and 337 > 123.

Given the upgrade of the laboratories’ LC-MS device during the study, samples from experiment 1 were partly (re-) measured on a Sciex TripleQuad 7500 equipped with an OptiFlow Pro Ion Source (Sciex, Framingham, MA, USA) coupled to a Shimadzu Nexera HPLC/UHPLC LC 40D XR (Shimadzu, Kyoto, Japan) using the same column and method. The acquisition method was optimized and checked against measurements on the old system to ensure comparability.

### 2.5. Untargeted LC-qTOF-MS Data Acquisition

Untargeted metabolomics analysis was conducted in randomized order on a Thermo Scientific Dionex UltiMate 3000 UHPLC system (Thermo Fischer Scientific, Waltham, MA, USA) coupled to a high-resolution time of flight (TOF) instrument (Sciex TripleTOF 6600; Sciex, Framingham, MA, USA) equipped with an IonDrive Turbo V source as described in detail elsewhere [19,20]. All samples were measured twice, once in ESI positive mode after reversed-phase (RP) chromatography on a XSelect^®^ HSS T3 2.5 µm, 2.1 × 150 mm XP column (Waters, Wexford, Ireland) and once in ESI negative mode following hydrophilic liquid interaction chromatography (HILIC) on a SeQuant^®^ ZIC^®^-HILIC 3.5 µm, 2.1 × 150 mm column (Merck, Darmstadt, Germany). The column oven was set to 40 °C and the injection volume to 3 µL, each. Eluents for RP were 10 mM ammonium formate with 0.1% (*v*/*v*) formic acid in water (A) and methanol with 0.1% (*v*/*v*) formic acid (B). Eluents for HILIC were 25 mM ammonium acetate with 0.1% (*v*/*v*) acetic acid in water (C) and acetonitrile with 0.1% (*v*/*v*) acetic acid (D). The RP gradient (0.5 mL/min, increased to 0.7 mL/min after 15 min) was 0–1 min 100% A, 1–15 min to 100% B, 15–18 min hold, 18.1 min return to starting conditions, 20 min re-equilibration. The HILIC gradient (0.5 mL/min) was 0–1 min 95% D, 1–10 min to 40% D, 10–12 min to 10% D, 12–13 min hold, 13 min return to starting conditions, 17 min re-equilibration [19].

### 2.6. Evaluation of Assay Performance in the Presence of Blood or Plasma (Experiment 1)

To evaluate the general influence of the presence of biological matrices on the CYP assay, the in vitro assay described under 2.3 was performed using HLM with the addition of the exogenous substrate mix (10 µM, n = 5 for buffer, whole blood, and plasma, respectively). In addition, negative controls Neg1 and Neg2 were incubated (n = 5 each), also including the exogenous probe substrate mix. Data acquisition was performed in targeted mode as detailed under 2.4. The CYP reactions of dextromethorphan (CYP2D6), omeprazole (CYP2C19), and midazolam (CYP3A4) were monitored through peak integration of the parent drugs and their corresponding metabolites dextrorphan, 5-hydroxyomeprazole, and 1-hydroxymidazolam in MultiQuant (Version 2.1.1296.0), respectively. The resulting peak areas were statistically compared between the assays performed in buffer and whole blood, as well as buffer and plasma, by a pairwise Wilcoxon rank-sum test (two-sided, *p* < 0.05). Additionally, peak areas of the following postulated endogenous CYP biomarkers were evaluated: serotonin to 5-methoxytryptamine for CYP2D6, arachidonic acid to 11,12-EET, 14,15-EET, 11,12-DiHET, and 14,15-DiHET for CYP2C19, and cortisol to 6-hydroxycortisol for CYP3A4, respectively.

### 2.7. Untargeted Analysis for Initial CYP Biomarker Search (Experiment 2)

We tested the feasibility of detecting CYP-dependent endogenous biomarkers using human liver microsomes (HLM) and isoenzymes (CYP2D6, CYP2C19, CYP3A4) in incubations (n = 5 each; prepared at the same time) with plasma as an endogenous substrate mixture (CYP reaction), compared to two matched plasma negative controls (Neg1, Neg2; n = 5 each; dataset 1). QC pools were prepared separately for the CYP reaction (pool CYP reaction) and negative controls (pool negative) by pooling 10 µL of each sample’s supernatant. Pools were diluted with buffer (100%, 80%, 60%, 40%, and 20%, *v*/*v*) to create diluted pools. The same setup was repeated with the addition of an exogenous substrate mix (100 nM) to each sample, serving as an in-assay positive control (dataset 2).

Data acquisition was performed using an untargeted LC-MS workflow as provided under 2.5. The resulting data files were analyzed using MS-DIAL (5.4.241004) [21] for peak picking and alignment, SIRIUS (Version 6.1.0) [22] for tentative compound annotation, and R (Version 4.5.1) [23] for data filtering and statistical analysis as detailed below. MS-DIAL parameters were adapted from Wartmann et al. [24]: profile mode, feature detection using Linear Weighted Moving Average smoothing (level 3), a minimum peak height of 1000, and a mass slice width of 0.05 Da. Deconvolution retained up to five isotopes per feature, with mass tolerances of 0.01 Da (MS1) and 0.04 Da (MS2). Alignment used a 0.05 min retention time tolerance and a 0.8 spectrum similarity, with gap filling applied. Other parameters, such as adducts, were left at default settings. Features were defined by retention time and mass-to-charge ratio (*m*/*z*). The resulting feature list was then further processed using SIRIUS. In SIRIUS, a maximum ppm error of 30 was allowed. Additionally, +H, ±H2O, +H3N, and +CH4O were allowed adducts in positive mode, while -H, ±H2O, and +ACN were allowed in negative mode. SIRIUS was configured with all ‘Bio’ databases queried, with PubChem and de novo as fallback. SIRIUS uses a combination of accurate mass, isotopic pattern, MS/MS fragmentation, and matching to public databases via its algorithm to identify features. Tentatively annotated features were classified according to the Metabolomics Standard Initiative (MSI) in confidence levels 1 (identified against a matching reference standard), level 2 (putatively annotated compound, e.g., against a public/commercial library), level 3 (putatively characterized compound class), and level 4 (unknown), respectively [25]. Peak areas exported from MS-DIAL from dataset 1 (without the exogenous substrate mix) were statistically evaluated for HLM and each isoenzyme in R. A linearity filter (eitherdiluted pool, r^2^ > 0.8, *p* < 0.05) removed non-linear features. A Kruskal–Wallis test was performed to assess differences among the three conditions (CYP reaction, Neg1, and Neg2). For features with a significant Kruskal–Wallis result (*p* < 0.05), post hoc pairwise comparisons were conducted using Dunn’s test with Holm correction. Global false discovery rate (FDR) control across all features was not applied. Only features significantly different between CYP reaction and both negative controls, but not between Neg1 and Neg2, were retained.

The remaining features were checked against samples containing the added exogenous probe substrate mix (dataset 2), where a visually similar (increase/decrease in mean peak area) or no trend in the difference between reaction and negative control samples was allowed. This ensured that features were altered reproducibly while maintaining the possibility of enzyme inhibition/occupation by the added exogenous substrates. Further, all features of interest were manually investigated for their trends (i.e., increase or decrease in peak area between the reaction assay and the negative control assays) across all isoenzymes. A feature was considered promising if it showed a consistent pattern (e.g., decreased in negative controls, increased in one or more CYP reaction conditions). Finally, all remaining features were manually checked in MS-DIAL to ensure no artifacts were kept (Peak shape, intensity across samples, gap filling, etc.). Features that might stem from the same entity but were separated by MS-DIAL were merged manually whenever possible (e.g., same retention time, *m*/*z*, adducts, MS/MS overlap/similar, etc.).

A feature was classified as a potential CYP substrate if its peak area decreased in the CYP reaction vs. negative controls. If peak area increased, it was classified as a potential metabolite. A fold change for each feature was calculated by dividing the median of the reaction condition by the mean of the median of both negative conditions.

Putative substrate–metabolite pairs were identified by matching selected features with mass differences of ±14.0157 Da (hydroxylation/demethylation) or ±15.9949 Da (epoxidation), within 30 ppm tolerance. To enable valid comparisons across ionization modes, each feature’s *m*/*z* was first converted to its neutral mass, assuming the most common adducts (typically [M+H]+ or [M-H]-). All putative substrate–metabolite pairs were filtered to retain only those with a biologically plausible metabolic relationship: a feature annotated as a potential metabolite was retained only if it could be linked to its paired compound via a mass shift consistent with hydroxylation, epoxidation or demethylation, and vice versa for potential substrates, ensuring that the transformation direction (e.g., substrate → metabolite) was chemically and biologically consistent. Final results were manually reviewed to correct split features and ensure consistency.

### 2.8. Further Evaluation of the Potential CYP Biomarker (Experiment 3)

To further characterize the tentatively postulated CYP biomarker from experiment 2, additional assay incubations were performed with varying amounts of plasma (endogenous substrate mix). The reaction was performed as described above in HLM and all three isoenzymes with 10%, 50%, or 100% plasma, respectively (n = 5 for each condition). All samples were analyzed using the untargeted LC-MS method described under 2.5. To calculate the resulting peak areas for the features selected from experiment 2, targeted data evaluation was performed using MultiQuant™. Comparisons were made regarding the amount of plasma in the assay by visual inspection of peak areas. Features were only further considered when they visually showed a linear or exponential relationship between peak areas and plasma amounts.

## 3. Results

### 3.1. Evaluation of the General CYP Assay Performance in the Presence of Blood or Plasma (Experiment 1)

We evaluated the general suitability of the standard HLM CYP assay by monitoring the formation of the drug metabolites of the well-established CYP probe substrates dextromethorphan (metabolite: dextrorphan, CYP2D6), omeprazole (metabolite: 5-hydroxyomeprazole, CYP2C19), and midazolam (metabolite: 1-hydroxymidazolam, CYP3A4), respectively. Peak areas obtained in the different assays using either whole blood or plasma as the endogenous substrate mixture were normalized to the mean peak area of the standard assay in buffer. Initial experiments with 2 additional conditions of 50:50 (*v*/*v*) blood/plasma and buffer did not show a significant difference to blood/plasma-only conditions and were therefore dropped from further experiments.

**Figure 1:**
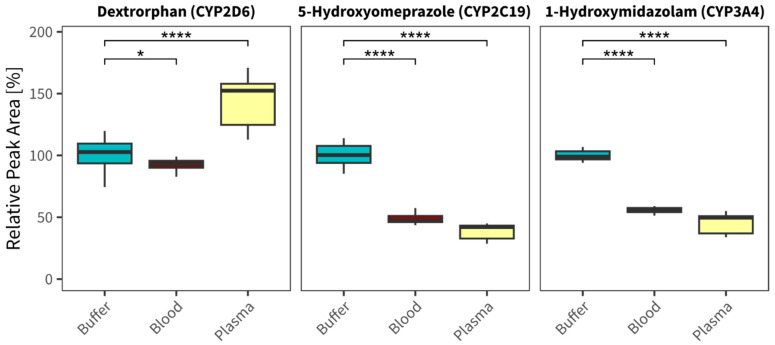
Boxplots of relative peak areas (normalized to buffer condition) of formed metabolites in HLM CYP assays in the presence of buffer (blue), whole blood (red), or plasma (yellow) (n = 15; n = 5 independent assay replicates, n = 3 technical replicates per independent assay replicate). Statistical comparison was performed using a pairwise Wilcoxon rank-sum test (* *p* ≤ 0.05, **** *p* ≤ 0.0001).

As depicted in Figure 1, statistically significant, but visually not relevant, slightly lower (whole blood) or higher formation (plasma) of dextrorphan was observed via CYP2D6. The CYP reactions of CYP2C19 and CYP3A4 performed similarly, although both enzymes exhibited significantly lower peak areas for 5-hydroxyomeprazole and 1-hydroxymidazolam, respectively, in the presence of both whole blood and plasma compared to the standard assay in buffer.

**Figure 2:**
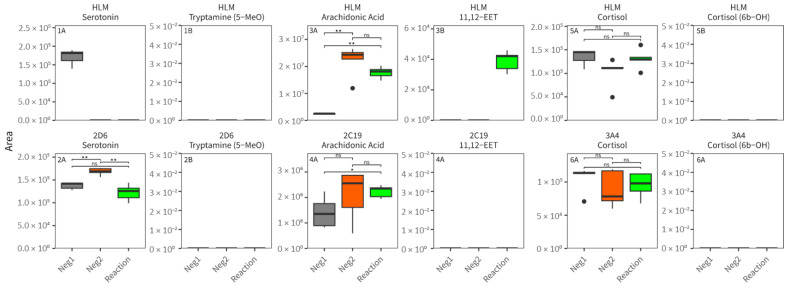
Proposed endogenous CYP substrates (**panels A**) and metabolites (**panels** B) are shown in HLM (top row, uneven panel numbers) and isoenzyme (bottom row, even panel numbers) assays (n = 5). Neg1 (gray), Neg2 (orange), and Reaction (green) conditions are shown on the x-axis. Peak areas are shown in the y-axis. Statistical comparison was performed using a pairwise Wilcoxon rank-sum test, not testing groups consisting of only 0 (* *p* ≤ 0.05, ** *p* ≤ 0.01, ns *p* ≥ 0.05).

In addition, as shown in Figure 2, endogenous CYP biomarkers postulated in the literature were evaluated in the presence of plasma in HLM and isoenzyme assays [3]. Serotonin, as a proposed CYP2D6 substrate, was readily metabolized in HLM assays; however, not only in the CYP reaction assay, but also in the absence of the CYP co-substrate (Neg2), which would omit the CYP reaction. CYP2D6 isoenzymes revealed a slight, but nonsignificant, decrease in serotonin concentrations in the CYP reaction assays. The expected metabolite 5-methoxytryptamine could not be detected, neither in HLM nor in CYP2D6 incubations. For CYP2C19, we found 11,12-EET and 14,15-EET, proposed metabolites formed from arachidonic acid, to be absent in the negative controls (Neg1 and Neg2, no CYP reaction expected), but present in high abundance in the HLM CYP reaction assay (due to similarity, only 11,12-EET is shown). In line with this, arachidonic acid levels were significantly lower in the CYP reaction compared to Neg2, but not Neg1. However, the same effects could not be observed in CYP2C19 isoenzyme incubations. 6β-hydroxycortisol formation, as a proposed indication for a CYP3A4 reaction, was not detectable in HLM and CYP3A4 isoenzyme incubations.

### 3.2. Untargeted Metabolomics Workflow for Tentative CYP Biomarker Search in In Vitro Assays (Experiments 2 and 3)

**Figure 3:**
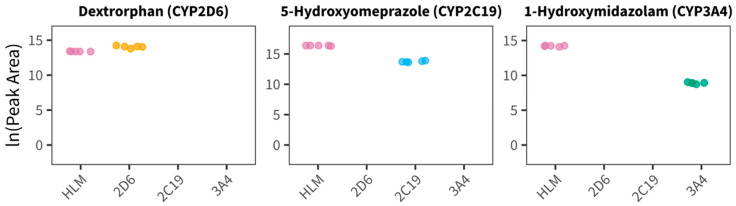
Comparison of drug metabolite peak areas (log transformed) for positive controls extracted by targeted search strategy (MultiQuant™) from untargeted data in all assays (HLM, CYP2D6, CYP2C19, and CYP3A4; n = 5 each).

An untargeted metabolomics workflow was applied to screen for potential new CYP biomarkers with the described in vitro assays. Data acquired by untargeted LC-MS/MS analysis following HLM and isoenzyme incubation in the presence of plasma vs. two different negative controls (no reaction by omitting either the enzyme or the co-substrate), were first submitted to a targeted data search (only dataset 2) for the expected metabolites dextrorphan, 5-hydroxyomeprazole, and 1-hydroxymidazolam as a positive control within the experiment. As depicted in Figure 3, we observed the formation of all three metabolites in HLM and isoenzyme assays, as expected. Additionally, other metabolites of the different probe substrates produced by different isoenzymes were identified among the different assays, e.g., 3-hydroxyomeprazole by HLM and CYP3A4, and 3-methoxymorphinan by HLM. While there seemed to be a potential trace of 3-methoxymorphinan in CPY3A4 assays, we are not confident enough to report it as a reliable finding.

Dextrorphan and 5-hydroxyomeprazole also appeared in our feature list after processing the data with MS-DIAL. However, the untargeted data processing workflow was unable to detect 1-hydroxymidazolam.

**Figure 4:**
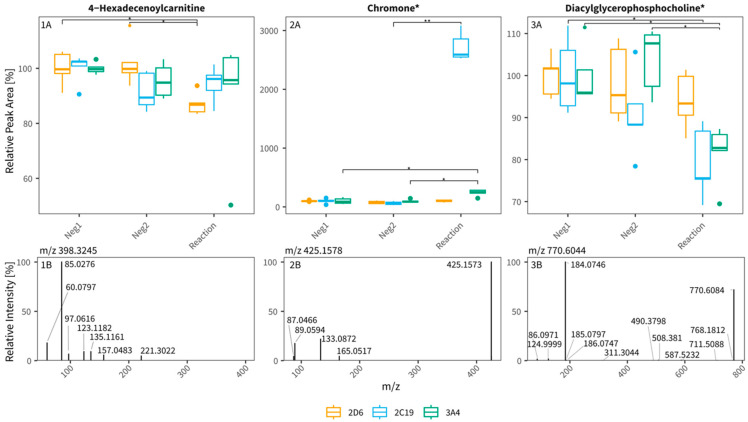
One potential exemplary feature per isoenzyme is shown (**panels 1**–**3**). The relative peak area (relative to Neg1) in each isoenzyme (CYP2D6, orange; CYP2C19, blue; CYP3A4, green) is shown on the y-axis (top row, **panels A**, n = 5) as well as the MS2 information of the features with *m*/*z* on the x-axis and relative intensity (relative to the highest peak) on the y-axis (bottom row, **panels B**). As in Table 1, proposed groups of compounds are indicated by * next to their name. All pre-cursor *m*/*z* are listed above the respective MS2 plot. Statistical comparison was performed using a Dunns-test with correction for multiple testing (Holm; * *p* ≤ 0.05, ** *p* ≤ 0.01).

**Figure 5:**
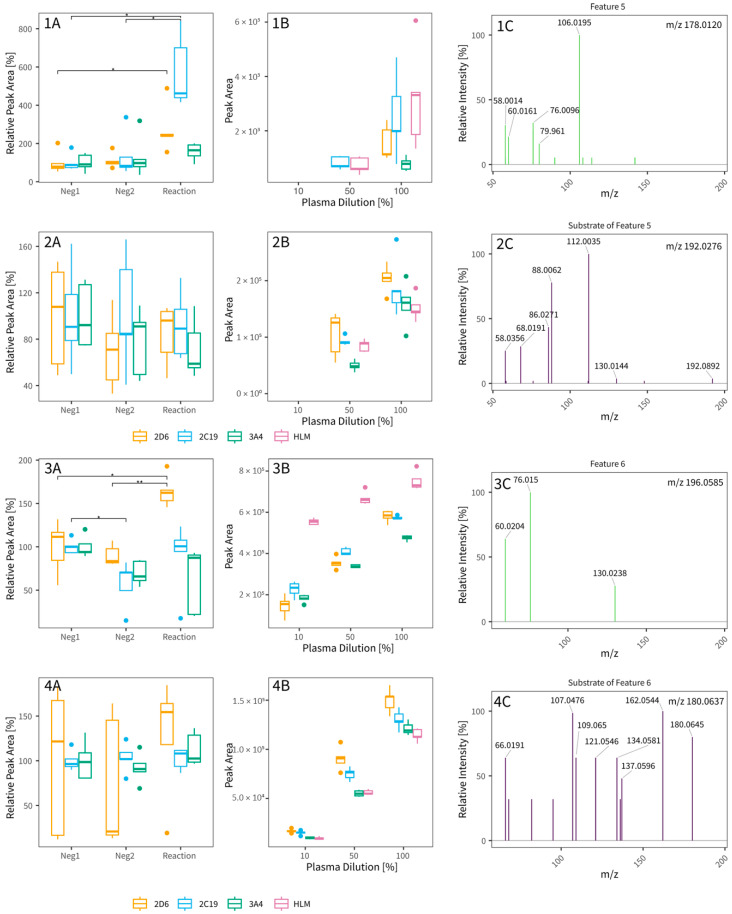
Two examples of postulated feature pairs are shown, each consisting of two features (Pair 1: 1: feature 5 according to Table 1, CYP metabolite; 2: corresponding substrate to 1, CYP re-action demethylation, CYP2C19; Pair 2: 3, feature 6 according to Table 1, CYP metabolite; 4, corresponding substrate to 3; CYP reaction hydroxylation, CYP2D6) For each compound, three panels (**A**–**C**) are presented: (**A**) relative peak area in the CYP reaction incubation and two negative controls across different isoenzymes, (**B**) peak area across plasma dilutions (**A**,**B**: n = 5), both shown as box-plots, and (**C**) the corresponding MS^2^ spectrum (green and purple lines respectively for pair partners). Isoenzymes are color-coded as follows: 2D6 (orange), 2C19 (blue), 3A4 (green), and HLM (pink). In the MS^2^ spectra, feature 5 (**panels 1A**–**C**) (or 6, **panels 3A**–**C**) is shown in lime green and its postulated pair partner (**panels 2A**–**C** or **4A**–**C**) in violet. All *m*/*z* values shown are neutral *m*/*z* calculated for comparison. Statistical comparison (**panels A**) was performed using a Dunns-test with correction for multiple testing (Holm; * *p* ≤ 0.05, ** *p* ≤ 0.01).

Comprehensive untargeted data processing in MS-DIAL, SIRIUS, and R to select potential CYP biomarkers from dataset 1 produced 28,708 and 11,995 features for RP+ and HILIC- data, respectively. After statistical filtering but before any manual inspection, 210 and 100 features remained for RP+ and HILIC-, respectively. Finally, after manual inspection and filtering based on the outcome of experiment 3 (matrix dilution), 29 features were retained as potential candidate CYP phenotype biomarkers, as summarized in Table 1. From these, 16 were classified as potential CYP substrates, as they showed lower peak areas in the CYP reaction assay compared to the negative controls (indicating substrate consumption), and 13 were classified as potential metabolites, with peak areas increased in the CYP reaction condition compared to the negative controls (indicating metabolite formation). In total, 16 features seemed to be specific for one isoenzyme, therefrom 10 for CYP2D6, 4 for CYP2C19, and 2 for CYP3A4, respectively. The 13 other features were metabolized by at least two enzymes (n = 10, 8 by CYP2D6 and CYP2C19, and 2 by CYP2D6 and CYP3A4, respectively) or by all three tested enzymes (n = 3). We were able to tentatively identify 19 features, 1 with a level 1, 3 with a level 2, and 13 with a level 3 classification (compound class only), according to the MSI. One example identification per isoenzyme is given in Figure 4. To improve identification, we evaluated the entire dataset for matching substrate–metabolite pairs of the most common reactions (hydroxylation, demethylation and epoxidation) and their corresponding mass spectral information. In total, we identified 10,936 potential metabolic relationships. After filtering for the 29 features of interest (Table 1), removing pairs with inconsistent transformation directions (e.g., metabolite to metabolite), and manually merging features that were split across peaks by MS-DIAL, 8 pairs remained with one feature (feature 6) accounting for two pairings as indicated in Table 1. Two example pairs (features 5 and 6) with their plots across different isoenzymes and incubation with different amounts of plasma are shown in Figure 5. While we have found a fitting substrate for feature 5 (feature demethylated) and a fitting substrate for feature 6 (feature hydroxylated/demethylated) based on assumed mass differences, both pair partners shown in Figure 5 do not readily behave as expected across isoenzyme reactions (e.g., a clear decrease in reaction for substrate partner of metabolite). Manual interpretation of the MS2 fragmentation pattern, where available, did not improve overall feature identification for any of the four examples.

Further information, such as isotope pattern and MS2 information, is given in Table S1 of the Supplementary Information. Further information on pairs is given in Table S2 of the Supplementary Information.

It is encouraging to see that, in our final feature list, many features still follow a linear or exponential behavior when adding higher or lower amounts of matrix (equivalent to lower or higher substrate concentrations), as shown in Figure S1 in the Supplementary Information.

## 4. Discussion

### 4.1. General CYP Assay Performance in the Presence of Blood or Plasma

Finding new endogenous CYP biomarkers has been attempted for several years, but common in vivo approaches are limited by ethical constraints, inter-individual variability, and small sample sizes per phenotype. In vitro approaches could partly overcome these limitations, particularly for an early-stage biomarker selection, but have never been evaluated for that purpose. Most importantly, a source of a large variety of human endogenous, potential CYP substrates needs to be added, which are not readily commercially available. Biological matrices could provide a natural substrate mix of human endogenous compounds, which we evaluated in HLM, as well as the most relevant drug-metabolizing CYP enzymes CYP2D6, CYP2C19, and CYP3A4, as a proof-of-concept.

Key differences between HLM and isolated isoenzymes must be acknowledged. While HLM represent not only drug-metabolizing CYP enzymes, but also the complete variety of (metabolic) enzymes biologically located in the endoplasmic reticulum, isoenzyme assays demonstrate high specificity. Isoenzymes are produced in a controlled manner, typically engineered from specific biological systems, ensuring a defined composition [26]. In contrast, HLM generally consist of pooled liver extracts from between 50 and 150 donors. Although information on donors is often available with the kits, this introduces variability from donor-specific factors such as age, sex, health status, and medication history. Beyond exogenous drugs, a vast array of endogenous compounds is inevitably introduced through the pooled HLM, e.g., fluctuations in arachidonic acid observed across assays (see Figure 2) [27,28,29,30]. Specificity is an important aspect of endogenous biomarkers. Hence, we generally focused on potential endogenous biomarkers found in our isolated isoenzyme assays, which would guarantee an involvement of the isoenzyme in that assay, acknowledging that such findings may not fully reflect in vivo dynamics but provide a critical starting point for targeted validation. Future work should test proposed compounds in increasingly complex in vitro systems or in authentic human in vivo samples.

A complex compound mixture like blood or plasma might lead to (unknown) inhibitory effects hindering the CYP reaction. Our initial experiments, therefore, included mixtures of blood and buffer (50/50, *v*/*v*) and monitored the CYP reaction of well-known probe substrates for the most relevant drug-metabolizing isoenzymes CYP2D6 (dextromethorphan to dextrorphan), CYP2C19 (omeprazole to 5-hydroxyomeprazol), and CYP3A4 (midazolam to 1-hydroxymidazolam). Our results showed minimal to no difference between using a 50:50 mixture of blood/plasma and buffer or blood/plasma only, hence we continued without the mixture condition. As shown in Figure 1, the CYP in vitro assays performed with adequate turnover rates, or even better (dextrorphan), when the buffer is replaced with blood or plasma. These results suggest plasma does not significantly impair CYP function under our conditions. Additionally, changing the blood anticoagulant (e.g., heparin instead of EDTA) may have an influence, as EDTA can bind divalent cations, such as MgCl_2_, which are needed for the enzymatic reaction. To mitigate this, we increased the MgCl_2_ concentration in the assay mix, which likely compensated for chelation. Future studies should explore other anticoagulants to further explore differences. We believe our experiments demonstrate that all investigated enzymes remain functional when the buffer is replaced with blood or plasma (using EDTA as a stabilizer). Although plasma is the standard matrix in clinical metabolomics, the use of whole blood in initial feasibility testing highlights a practical consideration: red blood cells and other cellular components may introduce variability or interfere with CYP activity. The decision to use plasma for screening was driven by its superior compatibility with standard LC-MS workflows and its established use in forensic and clinical settings. Hence, we continued our proof-of-concept study using plasma only. Only a few endogenous CYP biomarkers are described in the literature and are largely not clinically validated [3]. Still, we aimed to evaluate these in targeted analysis as a further proof-of-concept for the general performance of the CYP in vitro assay with biological matrices. From the postulated markers, we found serotonin, proposed as a biomarker for CYP2D6, only in our plasma sample incubations. Its disappearance in both HLM enzyme assays, with and without CYP co-substrates, however, suggests that other enzymes, besides CYP, are involved in the observed serotonin metabolism. The expected CYP metabolite, 5-methoxytryptamine, remained undetectable in these assays, despite sufficient method sensitivity for its detection. Although the peak areas were slightly lower compared to the negative control without co-substrates, potentially indicating serotonin consumption in CYP2D6 isoenzyme incubations, no clear trend could be observed. This finding also underscores the importance of running specific assays or a combination of such assays. With increasing complexity, such as HLM instead of specific isoenzyme assays, the potential for reactions outside the scope of interest increases significantly. In addition, we have found 11,12-EET, a metabolite previously speculated to be involved in CYP2C19 metabolism, in our HLM/plasma incubations. It was only detected in samples able to undergo CYP reactions. This can be interpreted as proof that our assay successfully introduces the precursor of 11,12-EET, most likely arachidonic acid, which we also found in decreased concentrations in the CYP reaction assay, compared to negative control samples without the co-substrate. However, the involvement of CYP2C19 in its isoenzyme assay appears to be either minimal or non-existent, suggesting that 11,12-EET may be a non-functional plasma biomarker for CYP2C19 phenotyping. For CYP3A4, we monitored cortisol and its postulated CYP3A4-dependent metabolite 6β-hydroxycortisol, but no significant cortisol reaction in both HLM and CYP3A4 isoenzyme assays were observed. The lack of sensitivity for 6β-hydroxycortisol suggests that low-abundance metabolites may be missed even on a highly sensitive (7500 QTrap) device. Depending on the substrate concentration in an assay and the turnover rate of the enzymes, measuring substrate decline rather than metabolite-formation can be insufficient to detect and characterize the enzymatic reaction.

### 4.2. Untargeted Metabolomics Workflow for Tentative CYP Biomarker Search in In Vitro Assays

We have found 29 statistically relevant features as potential candidate CYP biomarkers in our assays (Table 1), even after applying stringent statistical filtering and data cleaning, including a linearity filter to remove laboratory or instrumental contaminants and ensuring reproducibility across two sample sets. All three probe substrate metabolites (dextrorphan, 5-hydroxyomeprazole, and 1-hydroxymidazolam) were found by targeted processing of our untargeted acquired data, despite being 100-fold less concentrated than in our initial experiments, to avoid potential inhibition effects. Both dextrorphan and 5-hydroxyomeprazole were also found by our untargeted processing workflow using MS-DIAL. This proves that the expected true-positive features are found using our methodology. Unfortunately, 1-hydroxymidazolam was not detected following the untargeted workflow, even before any statistical analysis (raw feature list). This could be due to its very low abundance or other factors influencing peak detection, a known challenge in metabolomics as described in the literature [24]. While we were aware that our added exogenous probe substrates could alter the assay’s performance-hence prepared samples both with and without them-we cannot rule out the possibility that there might be analytes in our endogenous matrix that also alter/inhibit CYP3A4 to some degree despite the general evaluation of the assay exhibiting effective enzyme activity under the chosen conditions [29].

In general, when pivoting away from single or a select few substrate analyses, as commonly performed in CYP in vitro assays, ideal conditions for each endogenous compound present in the complex plasma matrix become impossible to achieve. While such optimizations are straightforward for known analytes, they are impractical for thousands of unknown endogenous compounds. Hence, it was clear that our assay would have to strike a middle ground, being ideal for many but not all reactions that occur. It is encouraging to see that, in our final feature list, many features still follow a linear or exponential behavior when adding higher or lower amounts of matrix (equivalent to lower or higher substrate concentrations), indicating that we have neither completely oversaturated nor diminished them. Obviously, this was certainly the case for other analytes that did not pass the final statistical filtering criteria, potentially due to being overabundant (thus statistically significant changes are impossible to observe), or their low concentration (not detectable with our method sensitivity, statistical parameters, or MS-DIAL).

Statistically, fold-changes in postulated metabolites overall were more pronounced compared to potential substrates. This observation is very reasonable, given that substrates might be overabundant and enzymes can only metabolize a fraction of them. Metabolites, on the other hand, may not be present at all or at very low concentrations. However, their production by the enzyme assay significantly increases their concentration, resulting in a substantial difference from the negative controls. This further explains why fold changes are higher for metabolites.

Some of the features were metabolized by more than one enzyme (Table 1). While availability, concentration, or barrier functions in vivo might limit these observations, it seems reasonable that some features are metabolized by multiple enzymes, especially substrates that could be altered to very different metabolites. For example, the formation of 11,12-EET in high concentrations in HLM, but not in isoenzyme incubations (CYP2D6, 2C19, 3A4), points to isoenzymes other than those investigated here. Typically, isoenzymes from the classes CYP2 or CYP4 are described as being responsible for the metabolism of fatty acids, steroids, or eicosanoids while CYP11, CYP17, CYP19, CYP21 and CYP27 specialize in steroid and cholesterol metabolism [31,32,33,34]. Sufficient isoenzyme specificity so far poses one of the significant concerns of endogenous CYP phenotyping, refs. [3,17] but could now be easily investigated with the described assay procedures.

Despite several potential biomarkers indicating the suitability of the proposed in vitro assays for this purpose, their unambiguous identification remains the primary bottleneck, as in any metabolomics study. To improve identification, we evaluated the entire dataset (not just the significant feature list) for matching substrate–metabolite pairs to the observed significant features of the most common reactions (hydroxylation, epoxidation and demethylation) and their corresponding mass spectral information. While 8 matching pairs based on their accurate mass differences were deduced, that unfortunately did not improve compound identification. This strategy could theoretically be extended to more metabolic reactions, but taking possible adducts into account as well, this also increases the number of potentially false-positive hits. The already high number of potential pairs (10,936) underscores the risk of false associations, which we address through manual curation and biologically plausible filtering. It is also worth considering that each pair found could be part of a metabolic chain we did not uncover. Ultimately, we could only suggest a handful of tentative identifications, with many only hinting at structural groups, with the most important ones briefly discussed in the following. This approach, while exploratory, offers a scalable framework for initial screening. We are aware that this general association is very prone to error and exploratory, yet wanted to demonstrate its potential.

Benzoic acid ester: Aromatic hydroxylation by CYP3A4, as well as O-dealkylation, are very likely reactions occurring regularly. Similarly, O-dealkylation by CYP2C19 is plausible as the enzyme handles a range of aromatic esters and ethers prominently, including omeprazole or fluoxetine as examples [35]. The involvement of CYP2D6 is a bit more obscure; although it can handle O-dealkylation, it usually prefers a basic nitrogen to be present for its reactions. Again, as we could not further elucidate the structure of this feature, it remains unclear if it is just a very favorable steric compound to also be involved with CYP2D6 or whether other factors contributing to this reaction. As the feature we found is supposed to be a metabolite, there might have been a basic nitrogen present in the reaction’s substrate [31].

Carnitines: Feature 14, tentatively identified as 4-hexadecenoylcarnitine, belongs to the class of acylcarnitines, a class of endogenous compounds previously described as novel urinary biomarkers for CYP3A activity. These medium-chain acylcarnitines are supposedly metabolized through ω- or ω-1-hydroxylation, and all metabolites are found in lower quantities after inhibition experiments [13]. These findings are consistent with our data, suggesting that 4-hexadecenoylcarnitine is a substrate for CYP enzymes. Inconsistent is the enzyme association, as we found it to be decreased in CYP2D6 isoenzyme reaction assays. There is a possibility that other enzymes, CYP3A4 specifically, could not metabolize enough of it to show a significant change in our statistics, as mentioned in the general limitations of analyzing substrates in this form of assay. Additionally, this study would be the first to find acylcarnitines altered by CYP isoenzymes in plasma.

Chromone: While chromone-like compounds (feature 17, 18) more likely originate from exogenous sources, CYP3A4 and CYP2C19 have been described to metabolize them [35]. Any chromone could be present in plasma due to the donor’s ingestion of sources containing it. While this would exclude it from the goal of finding an endogenous biomarker, it might still serve a purpose, as demonstrated by Magliocco et al. with solanidine, which is reliably present in the investigated population following regular potato ingestion [9].

Steroids: Steroid metabolism is a well-researched topic, with studies suggesting a role for CYP2C19 and CYP3A4 [36]. While CYP2D6 has been shown to metabolize steroids, its usual role is minimal and not largely observed in vivo due to other pathways handling those compounds [37]. We observed statistically significant formation of this feature (feature 16) in both CYP2D6 and CYP2C19 assays, but not in CYP3A4. As we were unable to elucidate the structure of this feature further, there is no clear indication why this might be the case. As described above, the simple nature of our assay, with little to no other enzymes and hence biological pathways available, could force the issue with CYP2D6 metabolism, otherwise not observed in vivo. Notably, this feature was not present in our HLM assays in the dilution experiment. This suggests instability or rapid further/different metabolism in complex systems.

Phospholipids: Phosphocholines, as well as oligo- or dipeptides, are not described to be metabolized by any CYP enzymes. Their apparent metabolism in our assay may reflect non-physiological conditions. Additionally, glycero-phosphocholines are often membrane bound which limits the in vivo access of CYP enzymes to them severely. As our assay does not mimic any biological barriers and inaccessibilities, there is a small possibility that this led to the metabolism of those features (features 21 and 29).

## 5. Conclusions and Outlook

In this proof-of-concept study, we could successfully show that our in vitro assay for metabolomics analysis of potential endogenous CYP450 phenotyping analytes works similarly well to established (iso)enzyme assays. The turnover of our substrates was well observed in buffer, blood and plasma. When using our assay to discover new endogenous CYP phenotyping markers, many potentially interesting features were found. Of those, a few could be tentatively identified albeit many only on a compound class level, proving that identification is still the largest bottleneck in metabolomics studies. While the employed TOF instrument proved the general possibility of performing in vitro assays with biological matrices in CYP biomarker search, a device with higher resolution might have improved identification capabilities. We believe that our assay is a great tool to facilitate metabolomics studies in fields that struggle to establish in vivo experiments for various reasons such as complexity, cost, or risk. Giving its easy laboratory handling, it could be extended to the simultaneous study of various CYP isoenzymes to screen for biomarkers with sufficient enzyme specificity. Future studies using pooled or multi-donor matrices could further assess biological variability and validate the proposed assay across populations. Ultimately, this work provides a foundational framework for future studies focused on developing accessible and efficient indirect CYP phenotyping methods. The approach is not intended to replace in vivo phenotyping but to enable preliminary screening of endogenous metabolites in controlled in vitro systems, which can then be validated in larger, diverse cohorts. Having a panel of well-described analytes that potentially could be looked at in a targeted manner or in simultaneous measurements with a much smaller in vivo study hugely benefits these endeavors.

## Figures and Tables

**Table 1:** All proposed features alongside the likely involved enzymes, their feature type and fold change, the analysis method they were acquired with, a proposed formula where possible as well as a potential identification with levels according to the Metabolomics Standards Initiative. Ref. [25] Identifications of level 3, compound class, are indicated with a * next to the name for clarity. Pair partner *m*/*z* differences are indicated as the potential relationship and rounded proposed difference in *m*/*z*.

ID	*m*/*z*	Time	Enzyme	Feature Type	Fold Change	Analysis	Proposed Formula	Proposed Ident.	Id. Level	Pair Partner (Neutral *m*/*z*)	Pair Prop. Formula
1	131.047	7.02	all	substrate	0.79	HILIC ESI-	C_4_H_8_N_2_O_3_	Asparagine	1	Metabolite, *m*/*z* 118.039, (∆= 14.0153)	C_3_H_6_N_2_O_3_
2	138.055	1.53	2D6	metabolite	1.42	HILIC ESI-	-	-	4		
3	165.076	4.23	2C19	metabolite	1.13	HILIC ESI-	C_6_H_14_O_5_	L-Fucitol	2	Substrate *m*/*z* 150.0886 (∆ = 15.9948)	C_6_H_14_O_4_
4	171.007	7.04	2D6/2C19	metabolite	14.64	HILIC ESI-	-	-	4		
5	177.005	7.02	2D6/2C19	metabolite	5.40	HILIC ESI-	C_6_H_2_N_4_O_3_	-	4	Substrate, *m*/*z* 192.0276, (∆ = 14.0156)	C_7_H_4_N_4_O_3_
6	195.051	6.69	2D6	metabolite	1.66	HILIC ESI-	C_6_H_12_O_7_	-	4	Substrates, *m*/*z* 180.0637/210.0742 (∆ = 15.9947/14.0157)	C_6_H_12_O_6_/C_7_H_14_O_7_
7	243.172	5.08	2C19	metabolite	1.63	HILIC ESI-	C_12_H_24_N_2_O_3_	Ile-Ile	2		
8	248.984	7.03	2D6/2C19	metabolite	15.06	HILIC ESI-	-	-	4		
9	334.309	14.8	2D6	substrate	0.40	RP ESI+	C_22_H_39_NO	N-acyl-amine *	3	Metabolite, *m*/*z* 349.2972 (∆ = 15.9951)	C_22_H_39_NO_2_
10	359.858	7.84	2D6/2C19	substrate	0.63	RP ESI+	-	-	4		
11	368.315	15.98	2D6	substrate	0.18	RP ESI+	C_22_H_41_NO_3_	N-acyl-amine *	3	Metabolite *m*/*z* 383.3020 (∆ = 15.9948)	C_22_H_41_NO_4_
12	374.192	8.43	2D6/2C19	metabolite	4.66	RP ESI+	-	Oligopeptide *	3		
13	382.986	7.01	2D6/2C19	substrate	0.49	HILIC ESI-	-	-	4		
14	398.325	14.61	2D6	substrate	0.87	RP ESI+	C_23_H_43_NO_4_	4-Hexadecenoyl-carnitine	2		
15	412.210	8.19	2D6	substrate	0.53	RP ESI+	-	-	4		
16	417.335	15.08	2D6/2C19	metabolite	4.06	RP ESI+	C_27_H_44_O_3_	3-oxo-delta-4-steroid	3	Substrate *m*/*z* 400.3335 (∆ = 15.9947)	C_27_H_44_O_2_
17	425.156	10.02	2C19	metabolite	107.53	RP ESI+	-	-	4		
18	425.158	10.3	2C19	metabolite	32.36	RP ESI+	C_24_H_24_O_7_	Chromone *	3		
19	447.060	8.17	all	metabolite	1.60	HILIC ESI-	C_20_H_16_O_12_	Benzoic acid ester *	3		
20	505.203	3.52	2D6	substrate	0.82	HILIC ESI-	-	-	4		
21	532.283	15.13	3A4	substrate	0.79	RP ESI+	-	Phospholipid *	3		
22	547.776	6.68	2D6/2C19	metabolite	2.51	HILIC ESI-	-	-	4		
23	564.437	16.48	2D6/3A4	substrate	0.90	RP ESI+	C_30_H_62_NO_6_P	Phosphatidyl-choline *	3		
24	576.401	16.03	2D6	substrate	0.82	RP ESI+	C_30_H_58_NO_7_P	Monoacylglycero-phosphocholine *	3		
25	578.421	16.24	2D6/3A4	substrate	0.88	RP ESI+	C_30_H_60_NO_7_P	Monoacylglycero-phosphocholine *	3		
26	659.516	4.79	2D6	substrate	0.68	HILIC ESI-	-	-	4		
27	770.604	18.05	3A4	substrate	0.81	RP ESI+	C_44_H_84_NO_7_P	Diacylglycero-phosphocholine *	3		
28	822.639	18.71	all	substrate	0.86	RP ESI+	C_48_H_88_NO_7_P	Diacylglycero-phosphocholine *	3		
29	831.562	16.24	2D6	substrate	0.56	RP ESI+	-	Phospholipid *	3		

## Data Availability

The original data presented in the study are openly available in MetaboLights at https://www.ebi.ac.uk/metabolights/MTBLS13325 or MTBLS13325.

## Supplementary Materials

The following supporting information can be downloaded at: https://www.mdpi.com/article/10.3390/metabo15120791/s1, Figure S1: Proposed features peak areas across plasma dilutions; Table S1: Features of interest with MS information; Table S2: Proposed feature pairs with MS information.
