# Supplementary material for: Assessing the Feasibility of In Vitro Assays in Combination with Biological Matrices to Screen for Endogenous CYP450 Phenotype Biomarkers Using an Untargeted Metabolomics Approach—A Proof of Concept Study
Source: Metabolites. 2025 Dec 12;15(12):791. doi: 10.3390/metabo15120791 (PMC12735286; doi:10.3390/metabo15120791)
Supplement: Supplementary file 1 [file metabolites-15-00791-s001.zip › metabolites-4023316-supplementary.pdf]

*Table S1: All proposed features alongside the likely involved enzymes, their feature type and fold change, the analysis method they were acquired with, a proposed formula where possible as well as a potential identification with levels according to the Metabolomics Standards Initiative (Sumner et al., 2007). Identifications of level 3, compound class, are indicated with a * next to the name for clarity. Pair partner m/z differences are indicated as the potential relationship and rounded proposed difference in m/z. MS1 isotopic spectrum as well as MS2 spectrum are given in the format of m/z:intensity.*

ID	m/z	Time	Enzyme	Feature Type	Fold Change	Analysis	Proposed Formula	SIRIUS	ms_ms_spectrum
1	131.047	7.02	all	substrate	0.79	HILIC ESI-	C4H8N2O3	Asparagine	52.01877:48 58.02898:586 68.01383:12 70.02872:614 71.01332:60 71.02415:178 72.00773:602 95.02430:165 96.00748:60 97.01285:24 113.03285:320 113.66691:12 114.01649:361 131.04262:24
2	138.055	1.53	2D6	metabolite	1.42	HILIC ESI-	-	-	77.96560:48 79.95614:36 80.96446:12 82.95921:12 97.95034:12 107.95425:24 108.01884:24 123.02902:88 137.96108:12 137.96894:60 137.98827:12 138.01334:24 138.05187:36
3	165.076	4.23	2C19	metabolite	1.13	HILIC ESI-	C6H14O5	L-Fucitol	55.01848:36 57.03353:36 59.01400:100 71.01244:76 73.02849:36 79.95394:24 85.02700:36 89.02269:12 102.96131:12 107.03024:12 121.02981:12 164.83173:36
4	171.007	7.04	2D6/2C19	metabolite	14.64	HILIC ESI-	-	-	78.95433:5495 79.99619:15
5	177.005	7.02	2D6/2C19	metabolite	5.40	HILIC ESI-	C6H2N4O3	-	105.01222:109
6	195.051	6.69	2D6	metabolite	1.66	HILIC ESI-	C6H12O7	-	59.01317:152 75.00772:237 129.01657:66
7	243.172	5.08	2C19	metabolite	1.63	HILIC ESI-	C12H24N2O3	Ile-Ile	82.06177:26 110.01706:59 130.07741:148 153.01912:143
8	248.984	7.03	2D6/2C19	metabolite	15.06	HILIC ESI-	-		78.95404:1329
9	334.309	14.8	2D6	substrate	0.40	RP ESI+	C22H39NO	N-acyl-amine*	93.06675:133 109.09952:39 121.09998:44 135.11677:51 149.12775:26 159.11589:22
10	359.858	7.84	2D6/C19	substrate	0.63	RP ESI+	-	-	70.06529:45 103.05287:23 120.08126:50 157.10129:15 195.12265:18 322.20094:23
11	368.315	15.98	2D6	substrate	0.18	RP ESI+	C22H41NO3	N-acyl-amine*	55.05331:234 67.05329:476 69.06811:173 81.06866:741 95.08437:635 105.06864:145 109.10046:420 111.07896:106 119.08484:195 123.11531:335 133.09926:227 149.13096:207 158.15356:86 165.16443:25 175.14790:566 193.15850:294 228.19579:253
12	374.192	8.43	2D6/2C19	metabolite	4.66	RP ESI+	-	Oligopeptide*	110.07042:103 136.06212:30 159.08551:23 170.09039:20 199.09223:20 251.13759:15
13	382.986	7.01	2D6/2C19	substrate	0.49	HILIC ESI-	-	-	70.02853:60 113.03262:401 131.04265:554
14	398.325	14.61	2D6	substrate	0.87	RP ESI+	C23H43NO4	4-Hexadecenoylcarnitine	60.07975:57 85.02764:318 97.06155:21 123.11818:29 135.11611:29 157.04832:18 221.30222:15
15	412.21	8.19	2D6	substrate	0.53	RP ESI+	-	-	104.10683:152 184.07499:136
16	417.335	15.08	2D6/2C19	metabolite	4.06	RP ESI+	C27H44O3	3-oxo delta-4-steroid	69.07028:33 81.06840:51 97.06560:141 107.08617:59 121.06649:78 135.11737:65 149.09825:76 159.11838:52 177.12781:123 185.13315:26 187.15073:28 189.16700:28 203.18173:38 215.13834:36 229.15931:34 241.15510:34 253.20192:44 269.19047:100 297.22216:29 325.24945:31
17	425.156	10.02	2C19	metabolite	107.53	RP ESI+	-	-	365.21137:12 423.79966:12 425.15692:499 425.22060:24 425.25929:12
18	425.158	10.3	2C19	metabolite	32.36	RP ESI+	C24H24O7	Chromone*	87.04656:12 89.05935:48 133.08719:60 165.05173:12 425.15731:276 425.22232:12
19	447.06	8.17	all	metabolite	1.60	HILIC ESI-	C20H16O12	Benzoic acid ester*	78.95784:17 128.03203:17 167.03902:26 193.05357:29 236.05497:48
20	505.203	3.52	2D6	substrate	0.82	HILIC ESI-	-	-	85.02685:22 113.02141:47 175.01980:29 285.17482:152 461.19701:75
21	532.283	15.13	3A4	substrate	0.79	RP ESI+	-	Phospholipid*	86.09726:50 104.10752:451 104.69732:3 105.06955:3 107.10706:3 125.00434:3 146.98681:4 184.07540:24 311.25755:5 402.29929:4 473.21271:208 497.35606:5 514.35414:5
22	547.776	6.68	2D6/2C19	metabolite	2.51	HILIC ESI-	-	-	131.07357:100 173.09043:59 193.06014:52 244.14765:19 312.10617:21 326.14974:15 347.16179:33 353.16090:33 495.24342:18 517.71890:54 532.72299:122 547.72319:202
23	564.437	16.48	3A4/2D6	substrate	0.90	RP ESI+	C30H62NO6P	Phosphatidyl-choline*	60.08086:110 86.09655:158 104.10764:2803 104.65943:15 111.11613:26 125.00004:159 136.06170:73 166.06385:38 184.07409:375 227.17833:15 240.11079:15 309.28663:19 472.35189:20 546.44243:46 562.68734:32
24	576.401	16.03	2D6	substrate	0.82	RP ESI+	C30H58NO7P	Monoacylglycerophospho-choline*	60.08080:29 81.07002:23 86.09529:27 104.10609:228 124.99749:31 139.10967:27 161.13125:17 173.12979:18 184.07362:299 223.16493:21 279.23926:20 291.22725:15 305.25427:18 428.36134:18 464.37364:18 493.39474:34 517.28528:31 558.39651:102
25	578.421	16.24	3A4/2D6	substrate	0.88	RP ESI+	C30H60NO7P	Monoacylglycerophospho-choline*	60.08083:52 81.06863:46 86.09690:113 104.10762:721 109.10213:36 124.99801:71 136.11408:36 139.11183:32 151.14404:20 155.11195:15 162.13334:23 166.06586:25 173.13322:23 184.07385:1082 186.12120:29 246.22060:23 258.11018:28 263.24527:21 279.23877:48 292.23677:20 320.27041:22 376.36699:45 392.35523:58 406.36441:24 406.37753:24 430.38209:21 501.33702:40 524.46500:48 560.41360:346 576.64048:17
26	659.516	4.79	2D6	substrate	0.68	HILIC ESI-	-	-	168.03797:270
27	770.604	18.05	3A4	substrate	0.81	RP ESI+	C44H84NO7P	Diacylglycerophosphocholine*	86.09707:278 124.99993:313 184.07460:18008 184.91607:25 185.07967:76 185.51503:21 185.81870:21 186.07472:61 186.25472:20 186.51622:15 186.87984:17 187.40790:15 187.58320:15 188.25158:15 311.30436:19 490.37981:20 508.38100:72 587.52315:76 711.50881:35 768.18123:37 770.60843:12930 773.96248:15
28	822.639	18.71	all	substrate	0.86	RP ESI+	C48H88NO7P	Diacylglycero-phosphocholine*	86.09644:194 124.99983:216 184.07437:13096 184.74797:22 185.08021:41 185.37226:15 185.53683:23 185.95298:20 186.33235:17 186.94722:15 510.40722:23 536.41040:54 571.49240:21 617.55577:85 639.53755:158 754.54057:33 763.53176:166 820.06259:34 821.73048:15 822.63813:11864 823.62581:42 824.30648:15
29	831.562	16.24	2D6	substrate	0.56	RP ESI+		Phospholipid*	184.07234:37 219.17328:276 311.30245:21 346.17203:34 564.34448:110 648.49688:31 772.52704:35

Table S2: All proposed pairs are shown with both features m/z, neutral m/z and retention time in minutes as well as the difference between the neutral m/z of both features (delta m/z) their relationship and the fold change of the pair partner (Fold Change Partner). MS2 spectrum of the pair partners are given, where available, in the format of m/z:intensity.

Feature 1 m/z	Feature 2 m/z	Feature 1 neutral m/z	Feature 2 neutral m/z	Ret. Time Feature 1	Ret. Time Feature 2	delta m/z	Relationship	Fold Change Partner	MS2 Feature 2
131.0467	119.0460	132.0540	118.0387	7.02	1.18	14.0153	Metabolite	1.10	58.05671:23
165.0761	151.0959	166.0834	150.0886	4.23	3.77	15.9948	Substrate	1.14	55.05370:24 67.05347:12 77.03964:24 81.07079:12 91.05665:24 109.05928:12 135.08549:12
177.0047	191.0203	178.0120	192.0276	7.02	14.27	14.0156	Substrate	0.86	57.02835:160 57.49740:12 67.01178:181 75.01928:12 85.01981:275 86.99896:493 110.65401:12 110.99618:633 129.00712:24 147.00997:12 191.08197:24
195.0512	181.0710	196.0585	180.0637	6.69	5.17	15.9947	Substrate	1.09	56.04786:12 67.02640:48 69.04356:24 83.05434:12 83.05862:24 96.05283:24 108.05490:74 108.06468:12 110.07227:48 122.05279:48 122.06184:48 122.06769:12 123.04452:12 135.06538:48 135.07657:12 137.07893:24 137.08939:12 138.06684:36 163.06164:75 181.07180:60
195.0512	209.0669	196.0585	210.0742	6.69	4.57	14.0157	Substrate	0.95	146.90589:12 148.88468:12
334.3094	350.3045	333.3021	349.2972	14.80	14.84	15.9951	Metabolite	0.35	-
368.3146	384.3093	367.3073	383.3020	15.98	14.61	15.9948	Metabolite	0.45	-
417.3355	399.3262	416.3282	400.3335	15.08	0.86	15.9947	Substrate	0.69	-

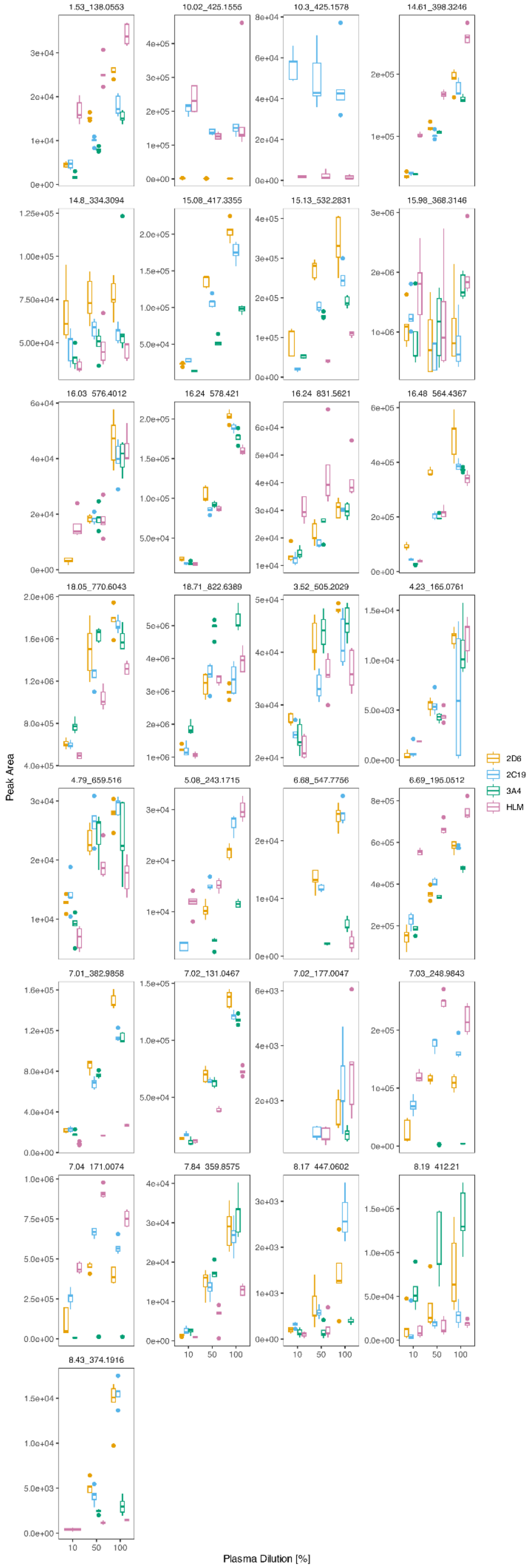

1.53_138.0553
10.02_425.1555
10.3_425.1578
14.61_398.3246
14.8_334.3094
15.08_417.3355
15.13_532.2831
15.98_368.3146
16.03_576.4012
16.24_578.421
16.24_831.5621
16.48_564.4367
18.05_770.6043
18.71_822.6389
3.52_505.2029
4.23_165.0761
4.79_659.516
5.08_243.1715
6.68_547.7756
6.69_195.0512
7.01_382.9858
7.02_131.0467
7.02_177.0047
7.03_248.9843
7.04_171.0074
7.84_359.8575
8.17_447.0602
8.19_412.21
8.43_374.1916
Peak Area
Plasma Dilution [%]
10
50
100
2D6
2C19
3A4
HLM

Figure S1: All proposed features peak areas across plasma dilutions are shown as boxplots, (Iso)enzymes are color-coded as follows: 2D6 (orange), 2C19 (blue), 3A4 (green), and HLM (pink).
